# Altered Cytokine Secretory Fingerprint of the Adipocytes Derived from Stem Cells of Morbidly Obese Patients—A Preliminary Study

**DOI:** 10.3390/cells13191603

**Published:** 2024-09-24

**Authors:** Bartłomiej Łukaszuk, Elżbieta Supruniuk, Adrian Chabowski, Agnieszka Mikłosz

**Affiliations:** Department of Physiology, Medical University of Bialystok, 15-089 Bialystok, Poland; elzbieta.supruniuk@umb.edu.pl (E.S.); agnieszka.miklosz@umb.edu.pl (A.M.)

**Keywords:** adipose tissue, stem cells, ADMSCs, cytokines, metabolic syndrome, IL-6

## Abstract

*Context:* Adipose-derived mesenchymal stem cells (ADMSCs) are progenitor cells that shape the tissue’s biological properties. *Objective:* To examine the adipocytes differentiated from the ADMSCs of lean and obese individuals with/without a metabolic syndrome (MetSx) cytokine secretory profile, as to date, little is known on this topic. *Methods:* Interleukin, chemokine and growth factor levels in the culture medium were determined using the Human Cytokine kit. *Results*: We observed a characteristic secretory fingerprint displayed by the cells from the MetSx group and identified a set of putative markers (IL-1β, IL-6, IL-7, IL-10, IL-12, IL-13, VEGF, FGF, GM-CSF, TNF-α, IFN-γ) of the condition. Surprisingly, the concentrations of most of the molecules (except for IL-6, IFN-γ, IP-10, VEGF) decreased when compared with the cells from the lean group. We postulate that the difference stemmed from the fact that in vivo cytokines were mostly secreted by the activated monocytes/macrophages and not adipocytes per se. This may also suggest that the aforementioned upregulated cytokines (IL-6, IFN-γ, IP-10, VEGF) might have been the ones that attracted monocytes and triggered the vicious cycle of tissue inflammation. *Conclusions*: Our study indicated that the adipocytes newly derived from the ADMSCs of obese patients with metabolic syndrome displayed a secretory fingerprint that may be characteristic to the early stages of the condition.

## 1. Introduction

Obesity, which is defined as an abnormally high body fat mass, is a metabolism homeostasis disturbance that stems from a chronic oversupply of nutrients [1]. The excessive energy substrates are stored within fat tissue, mainly in the form of triacylglycerols. Obesity is a key risk factor for noncommunicable diseases, like atherosclerosis, metabolic syndrome or type 2 diabetes, which contribute to a significantly reduced life span (~13 years for males and ~8 years for females) of the affected people [2]. Unfortunately, literature data on the condition prevalence are quite alarming. In 2015–2016, in the USA alone, roughly 40% of population was classified as obese (BMI ≥ 30 kg/m^2^) and circa 70% as overweight (BMI > 25 kg/m^2^) [3]. As of February 2024, the World Health Organization estimated that there were over 1 billion obese individuals worldwide and the number is expected to grow in the near future [4]. Unsurprisingly, the remarkable growth in the obesity pandemic since the 1970s has spawned a few decades of scientific research on the etiology of the condition.

Adipose tissue is present throughout the body, and its two most important depots are localized subcutaneously and within the visceral compartment [5]. In lean, healthy individuals, subcutaneous adipose tissue (SAT) constitutes ~80% of the tissue mass and fulfills the important thermoregulatory function of an insulator that prevents heat loss [6]. Moreover, it is a major source of circulating lipids, while its expansion via hyperplasia (generation of new fat cells) or hypertrophy (expansion of the mature adipocytes) serves as a ‘metabolic sink’ in the state of body lipid overload [7]. On the other hand, a person that maintains a proper body mass is characterized by a relatively small amount of visceral fat (VAT), which is significantly increased in obese individuals. VAT is considered to be the more metabolically active of the two depots, and due to its location, it impacts the abdominal organs (like the liver by fatty acids released into portal circulation) [8]. Importantly, hypertrophied adipocytes also release a set of adipokines that contribute to inflammation and shift the obesity toward metabolic syndrome and organ tissue complications [7]. In line with that notion, Bradley et al. demonstrated that murine 3T3-L1 adipocytes incubated for 24 h with palmitic acid (C16:0) displayed a ~70% increase in TNF-α (a proinflammatory cytokine) and a ~75% drop in IL-10 production (an anti-inflammatory cytokine) [9]. Furthermore, an elevated BMI is positively correlated with both the serum concentration and adipose tissue expression of IL-2, IL-6, IL-8, MCP-1 and other pro-inflammatory molecules [8]. Moreover, their levels are believed to underline the low-grade chronic inflammation present in obesity, which, in turn, is implicated in the development of cardiovascular diseases [8], asthma [10] or even vulnerability to a severe course of COVID-19 infection [11].

Interestingly, although adipocytes compose the vast majority (~90%) of the tissue volume, they constitute only approximately one third of the cells found in the fat pads. The remaining parts are represented by fibroblasts, endothelium, immune and stromal cells, as well as pre-adipocytes [8]. Adipose-derived mesenchymal stem cells (ADMSCs) are a set of progenitor cells present in the tissue and characterized by their multilineage differentiation potential. They serve as a birthplace of new adipocytes and, therefore, are an important factor that shapes the tissue’s biological properties. Moreover, investigators began to recognize them as a feasible treatment for a number of medical conditions, including the micro- and macrovascular complications of diabetes mellitus [12]. MSCs derived from obese patients displayed lowered migration capacity, mobilization and pro-angiogenic activity compared with the stem cells from lean donors [13]. Interestingly, our recent studies demonstrated [14,15] that the phenotype of mature adipocytes freshly derived from ADMSCs is influenced by both the origin place (SAT vs. VAT) and metabolic status of the patients. This was especially evident in the adipocytes that stemmed from the ADMSCs of obese patients with metabolic syndrome [14,15]. Yet, to date, little is known about the secretory profile of the adipocytes differentiated from ADMSCs. However, the examination of ‘freshly derived adipocytes’ would be of particular interest since studies demonstrated that the adipokines secreted by the tissue in vivo may, e.g., trigger the onset of insulin resistance in the heart, skeletal muscle [16] or liver [17]. Therefore, here we aimed to fill the gap in the literature data and deepen our understanding regarding the secretory profile of the mature adipocytes differentiated from ADMSCs. To this end, in the current study, we deployed the previously established protocol [14]. We aimed to thoroughly examine the concentration of cytokines released to the culture media by the adipocytes of sub- and visADMSC origins obtained from individuals with different metabolic statuses (lean, obese with and without the accompanying metabolic syndrome). Moreover, we were interested to know whether the cells preserved the characteristic secretory pattern observed in the three patient types in vivo.

## 2. Materials and Methods

### 2.1. Patients

Subcutaneous (abdominal region) and visceral (omental region) adipose tissues were collected from postmenopausal female patients who underwent an elective laparoscopic cholecystectomy or elective bariatric surgery at the First Department of General and Endocrine Surgery at the University Hospital in Białystok. The major exclusion criteria were an acute inflammatory process and a history of malignancy. The patients were divided into three groups: lean (n = 8), morbidly obese without accompanying metabolic syndrome (n = 8) and morbidly obese with metabolic syndrome (n = 8). The presence of metabolic syndrome was ascertained based on the following traits: (1) waist girth > 89 cm; (2) blood triacylglycerol concentration ≥ 150 mg/dL; (3) blood HDL cholesterol concentration <50 mg/dL; (4) blood pressure ≥ 130/85 mmHg; and (5) fasting blood glucose concentration ≥ 100 mg/dL. A female patient that displayed at least three of the above characteristics was diagnosed as positive for metabolic syndrome according to the National Institutes of Health’s criteria [18]. This study was approved by the Ethics Committee of the Medical University of Bialystok (permission no. R-I-002/187/2017) and was carried out in accordance with the Helsinki Declaration and the Guidelines for Good Clinical Practice. All patients that participated in this study gave their informed consent. The patients’ basic clinical data are available in Table S1.

### 2.2. Isolation, Cultivation and Characterization of Human Adipose-Derived Mesenchymal Stem Cells

Mesenchymal stem cells were isolated in accordance with a previously described protocol [19]. Briefly, adipose tissue samples were washed in phosphate-buffered saline (PBS, PAN-Biotech, Aidenbach, Germany) followed by digestion with collagenase at 37 °C for 1 h (250 U/mL collagenase NB 4G Proved Grade, Serva, Heidelberg, Germany). After the removal of mature adipocytes, the pellet was centrifuged for 10 min at 600× *g*, suspended in erythrocyte lysis buffer (Thermo Fisher Scientific, Waltham, MA, USA), and filtered through 200 µm and then 20 µm strainers. Following a second centrifugation, the pellet that contained ADMSCs was suspended in the culture medium (MSCM, ScienCell Research Laboratories, Carlsbad, CA, United States). The cells were cultivated until they reached 80–90% confluence. The ADMSCs were then counted, cryopreserved in a freezing medium (Stem-Cell banker DMSO Free, Takara Bio, Mountain View, CA, United States) and stored at −80 °C. The phenotype of ADMSCs and their multilineage differentiation potential were confirmed using flow cytometry and the Human Mesenchymal Stem Cell Functional Identification Kit (R&D Systems, Inc., Minneapolis, MN, USA), as described previously [19]. The isolated ADMSCs were stained using fluorochrome-conjugated monoclonal antibodies: anti-CD73, anti-CD90, anti-CD105, anti-CD45, anti-CD133 and anti-Lineage Cocktail 1 (CD3, CD14, CD16, CD19, CD20, CD56) (BD Bioscience, Franklin Lake, NJ, USA) in order to confirm their phenotype by means of flow cytometry [19].

### 2.3. Adipogenic Differentiation of ADMSCs

ADMSCs from 2 to 4 passages were seeded in MSCM culture medium and, after reaching confluence, adipogenesis was induced using a differentiation medium that contained supplements (MADM, Mesenchymal Stem Cell Adipogenic Differentiation Medium, ScienCell Research Laboratories, Carlsbad, CA, USA), 5% FBS and 1% penicillin/streptomycin solution. The progress of adipogenesis was monitored by the microscopic observation of lipid vacuoles in cells and Oil Red-O staining (Figure S1). The differentiation process took an average of 2 weeks, and mature adipocytes were used for further analysis.

### 2.4. Determination of the Levels of Cytokines, Chemokines and Growth Factors

The concentration of cytokines, chemokines and growth factors in the culture medium of ADMSC-derived adipocytes was determined using the Bio-Plex Pro Human Cytokine 27-Plex Assay (Bio-Rad Laboratories, Inc.; Hercules, CA, USA). Prior to the analysis, medium normalization was performed. Briefly, ADMSCs were seeded at approximately the same density (~20,000 cells per 6-well plate). Each well of each plate was provided with an equal amount of culture medium (1 mL). The cells were differentiated into adult adipocytes for 14 days. For the secretome analysis, the cells were maintained in a serum-free medium for 24 h in order to avoid the variability introduced by serum components. After that time, the medium was collected and the secretome analysis was conducted using a Human Cytokine Assay from Bio-Rad (cat no. #M500KCAF0Y) according to the manufacturer’s instructions. The assay measured the levels of the following cell-signaling molecules: basic fibroblast growth factor (bFGF), eotaxin, granulocyte-colony stimulating factor (G-CSF), granulocyte-macrophage colony stimulating factor (GM-CSF), interferon-gamma (INF-γ), interleukin-1β (IL-1β), interleukin 1ra (IL-1ra), interleukin-2 (IL-2), interleukin-4 (IL-4), interleukin-5 (IL-5), interleukin-6 (IL-6), interleukin-7 (IL-7), interleukin-8 (IL-8), interleukin-9 (IL-9), interleukin-10 (IL-10), interleukin-12 (IL-12), interleukin-13 (IL-13), interleukin-15 (IL-15), interleukin-17 (IL-17), interferon-gamma inducible protein, CXCL10 (IP-10), monocyte chemoattractant protein-1 (MCP-1), macrophage inflammatory protein 1 α (MIP-1α), macrophage inflammatory protein 1 β (MIP-1β), platelet-derived growth factor (PDGF), RANTES, tumor necrosis factor-α (TNF-α) and vascular endothelial growth factor (VEGF). Briefly, the cell culture medium was centrifuged at 1000× *g* for 15 min at 4 °C, and undiluted samples were used for the assay. In the first step, 50 µL of coupled magnetic beads were added to each well and the plate was washed twice with a 1 × assay buffer. Thereafter, 50 µL of standards, samples and controls were added to the appropriate wells of the plate, and the plate was incubated on a shaker at 850 rpm for 30 min at room temperature. After incubation, the plate was washed three times. Then, 50 µL of streptavidin–phycoerythrin (SA–PE) was added to each well and incubated for 10 min at room temperature and then washed again. Finally, the beads were resuspended in 125 µL of assay buffer and the plate was shaken at 850 rpm for 30 s. Analyte concentrations were calculated by taking into account the appropriate standard curve and expressed in pg/mL.

### 2.5. Statistical Analysis

**Figure 1:**
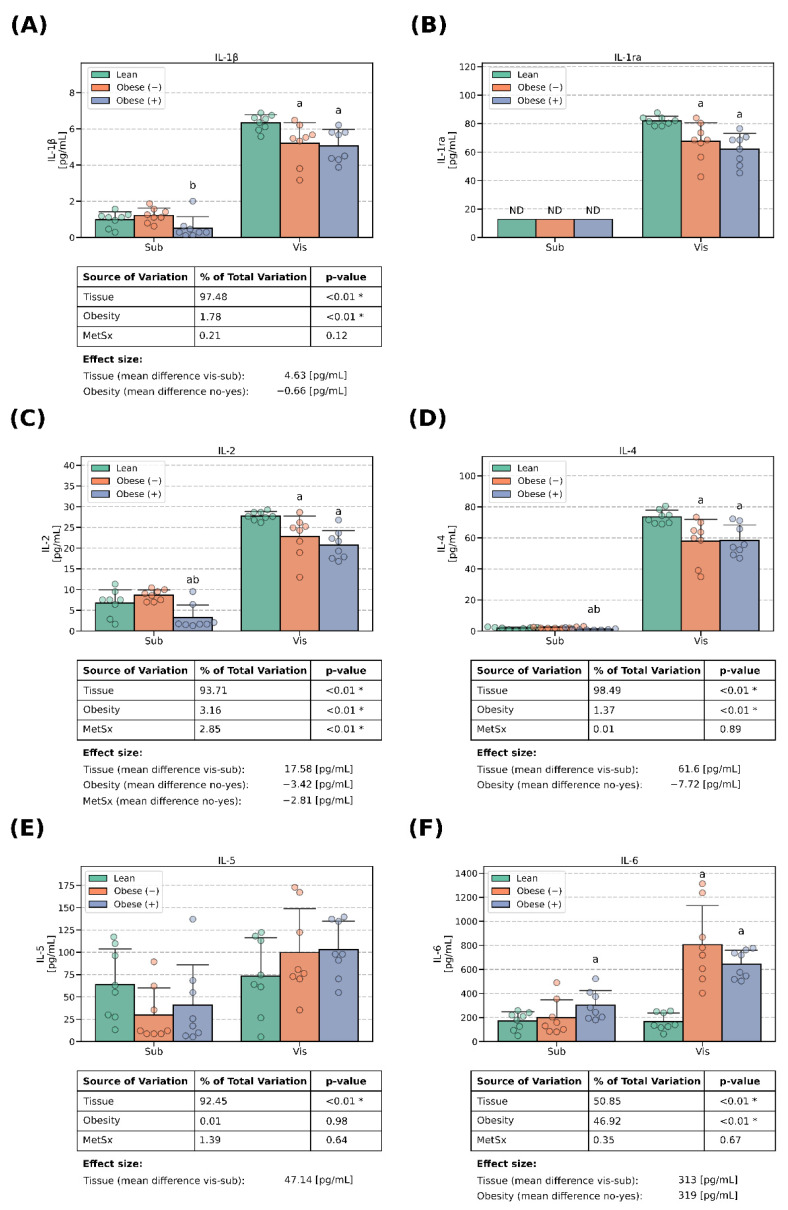
Interleukins (IL-1B, IL-1ra, IL-2, IL-4, IL-5, IL-6) in the culture media that bathed mature adipocytes differentiated from ADMSCs of subcutaneous and visceral provenances: (**A**) concentrations of IL-1ra, (**B**) concentrations of IL-1β (**C**) concentrations of IL-2, (**D**) concentrations of IL-4, (**E**) concentrations of IL-5 and (**F**) concentrations of IL-6. Data are depicted as the mean (bar height) and standard deviation (whiskers) calculated for n = 8 different samples per group. a: difference vs. lean (*p* < 0.05) within tissue type, b: difference vs. obese (−) (*p* < 0.05) within the tissue type. Obese (−): adipocytes differentiated from ADMSCs of patients with obesity but without metabolic syndrome, obese (+): adipocytes differentiated from ADMSCs of patients with obesity with accompanying metabolic syndrome. *—statistical significance (*p* < 0.05) of the examined factor in three-way ANOVA analysis.

**Figure 2:**
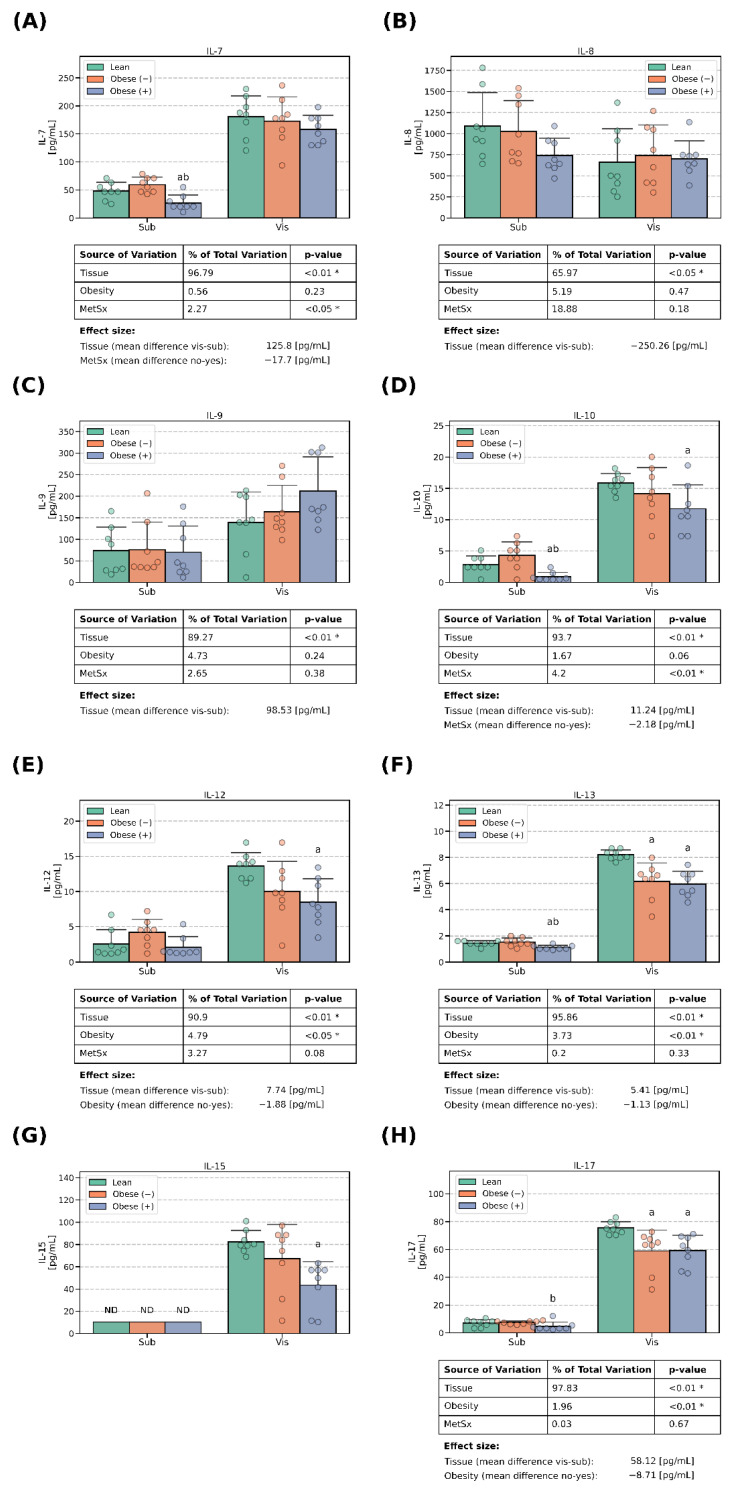
Interleukins (IL-7, IL-8, IL-9, IL-10, IL-12, IL-13, IL-15, IL-17) in the culture media that bathed mature adipocytes differentiated from ADMSCs of subcutaneous and visceral provenances: (**A**) concentrations of IL-7, (**B**) concentrations of IL-8, (**C**) concentrations of IL-9, (**D**) concentrations of IL-10, (**E**) concentrations of IL-12, (**F**) concentrations of IL-13, (**G**) concentrations of IL-15 and (**H**) concentrations of IL-17. Data are depicted as the mean (bar height) and standard deviation (whiskers) calculated for n = 8 different samples per group. a: difference vs. lean (*p* < 0.05) within tissue type, b: difference vs. obese (−) (*p* < 0.05) within the tissue type. Obese (−): adipocytes differentiated from ADMSCs of patients with obesity but without metabolic syndrome, obese (+): adipocytes differentiated from ADMSCs of patients with obesity with accompanying metabolic syndrome. *—statistical significance (*p* < 0.05) of the examined factor in three-way ANOVA analysis.

**Figure 3:**
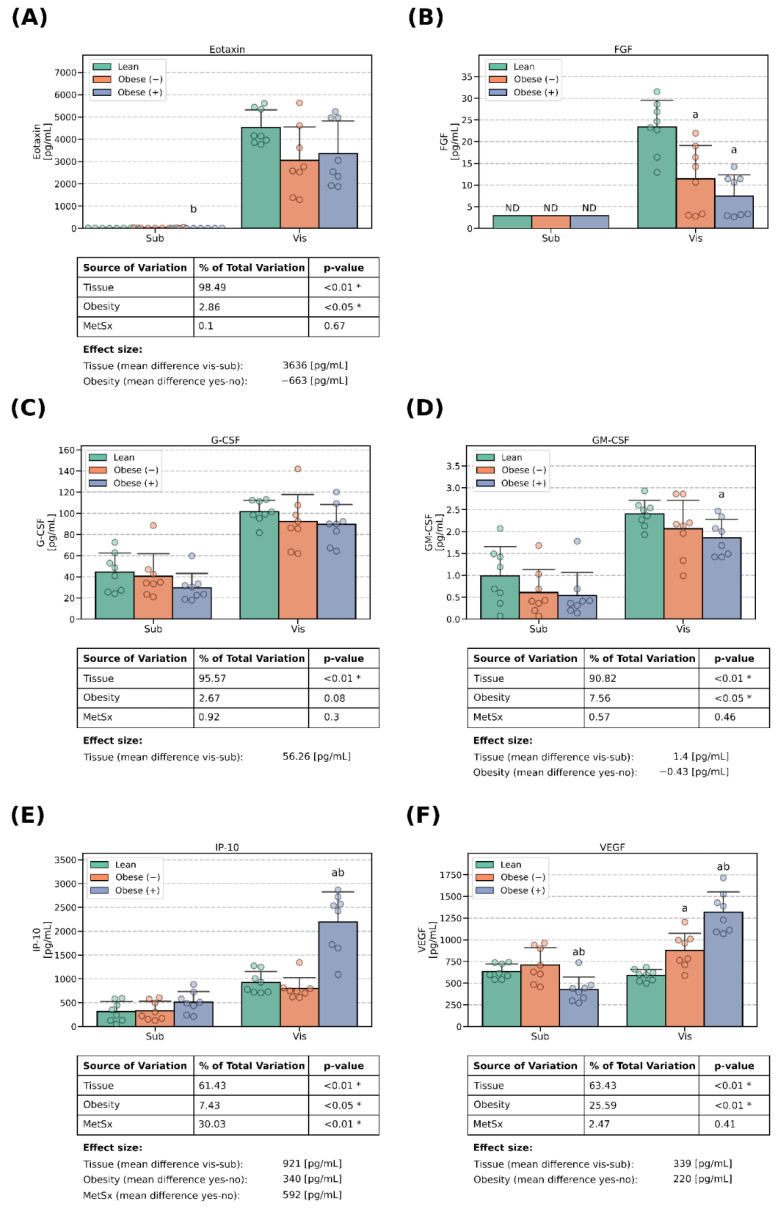
Growth factors in the culture media that bathed mature adipocytes differentiated from ADMSCs of subcutaneous and visceral provenances: (**A**) concentrations of Eotaxin, (**B**) concentrations of FGF, (**C**) concentrations of G-CSF, (**D**) concentrations of GM-CSF, (**E**) concentrations of IP-10 and (**F**) concentrations of VEGF. Data are depicted as the mean (bar height) and standard deviation (whiskers) calculated for n = 8 different samples per group. a: difference vs. lean (*p* < 0.05) within tissue type, b: difference vs. obese (−) (*p* < 0.05) within the tissue type. Obese (−): adipocytes differentiated from ADMSCs of patients with obesity but without metabolic syndrome, obese (+): adipocytes differentiated from ADMSCs of patients with obesity with accompanying metabolic syndrome. *—statistical significance (*p* < 0.05) of the examined factor in three-way ANOVA analysis.

**Figure 4:**
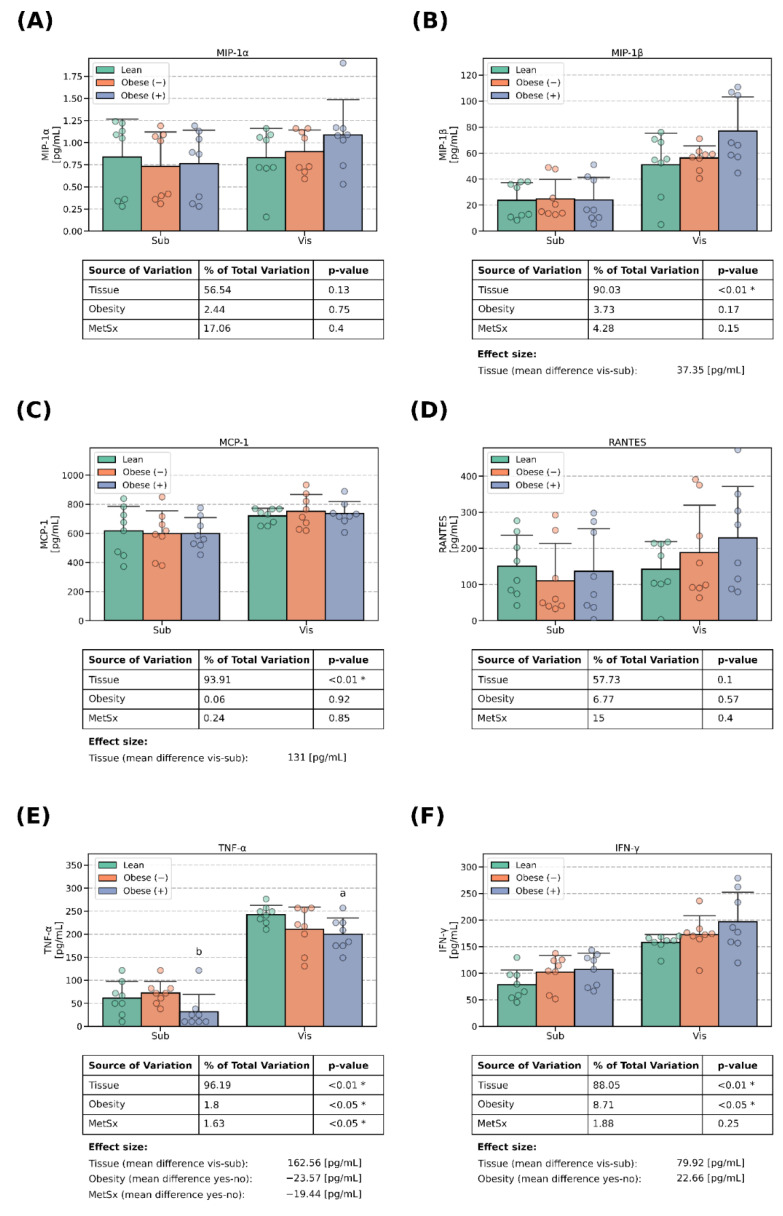
Chemokines in the culture media that bathed mature adipocytes differentiated from ADMSCs of subcutaneous and visceral provenances: (**A**) concentrations of MIP-1α, (**B**) concentrations of MIP-1β, (**C**) concentrations of MCP-1, (**D**) concentrations of RANTES, (**E**) concentrations of TNF-α and (**F**) concentrations of IFN-γ. Data are depicted as the mean (bar height) and standard deviation (whiskers) calculated for n = 8 different samples per group. a: difference vs. lean (*p* < 0.05) within tissue type, b: difference vs. obese (−) (*p* < 0.05) within the tissue type. Obese (−): adipocytes differentiated from ADMSCs of patients with obesity but without metabolic syndrome, obese (+): adipocytes differentiated from ADMSCs of patients with obesity with accompanying metabolic syndrome. *—statistical significance (*p* < 0.05) of the examined factor in three-way ANOVA analysis.

**Figure 5:**
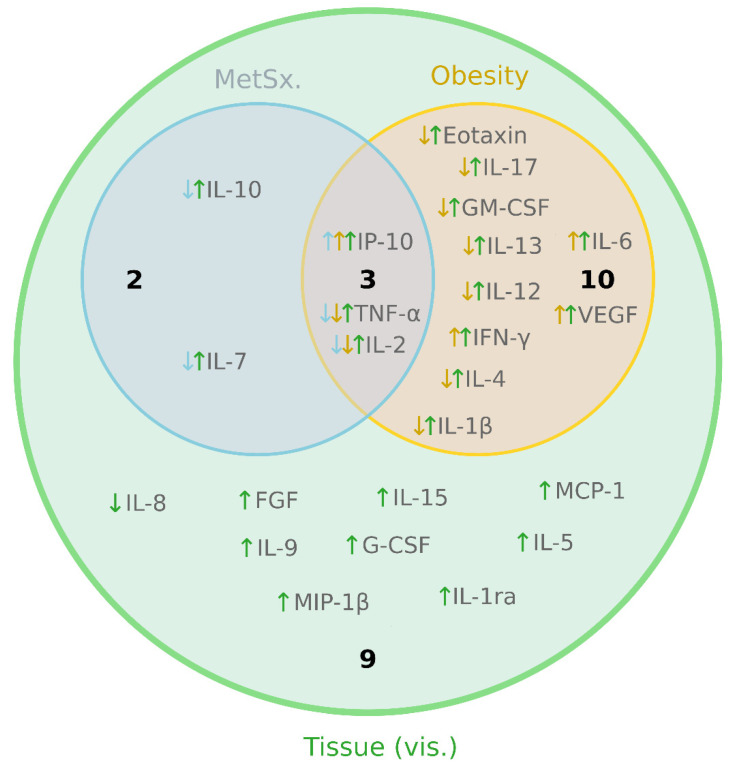
Venn diagram depicting the relationships between the examined factors (tissue, obesity and metabolic syndrome). The numbers in the circles depict the number of investigated molecules (Figure 1, Figure 2, Figure 3 and Figure 4) affected by a given factor. The arrows alongside a molecule name indicate the direction of a change exerted by a factor. The color of an arrow corresponds with the color that represents an analyzed factor.

**Figure 6:**
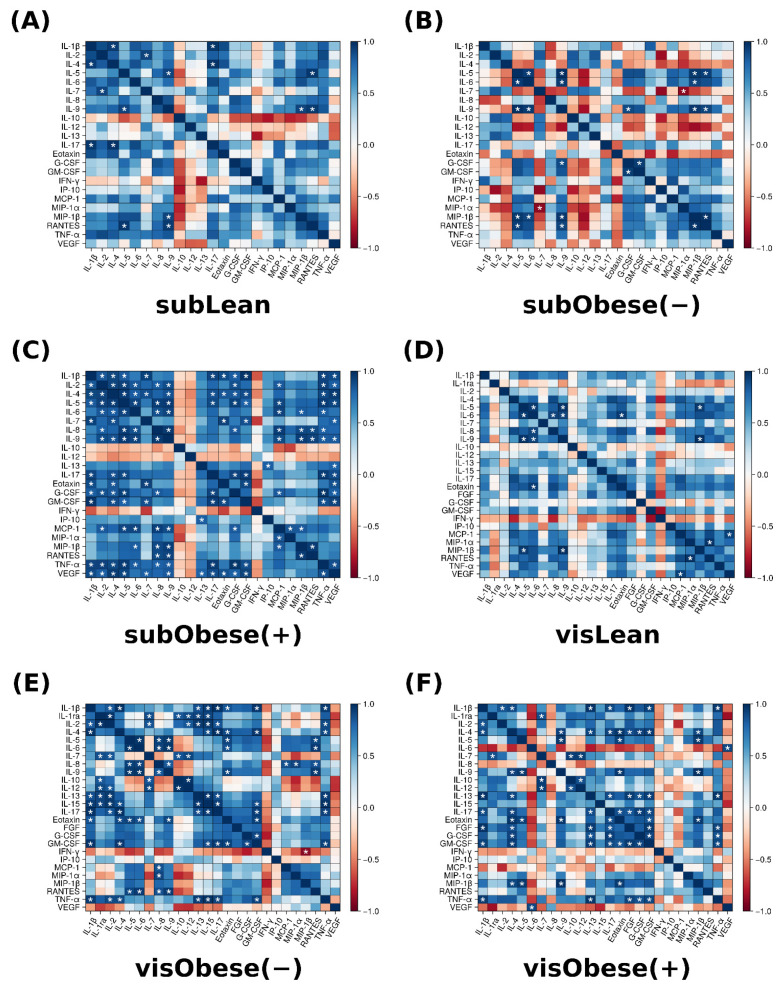
Pearson correlation coefficients depicted as heatmaps for the (**A**) subLean (**B**) subObese (−), (**C**) subObese (+), (**D**) visLean, (**E**) visObese (−) and (**F**) visObese (+) groups. Positive correlations are expressed as shades of blue, while negative correlations correspond to the shades of red. Correlations were calculated for n = 8 different samples per group; *: statistically significant (*p* < 0.05) correlation. Obese (−): adipocytes differentiated from ADMSCs of patients with obesity but without metabolic syndrome, obese (+): adipocytes differentiated from ADMSCs of patients with obesity with accompanying metabolic syndrome.

**Figure 7:**
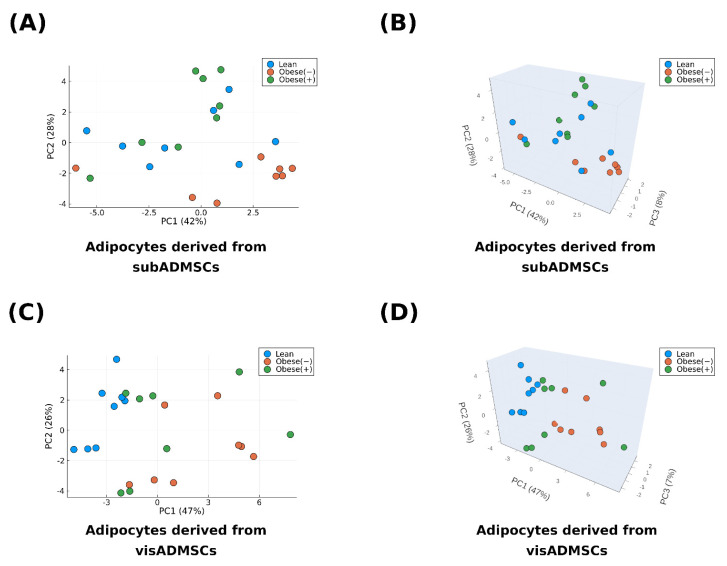
Graphical depictions of the principal component analysis for the cytokines secreted by (**A**,**B**) adipocytes derived from subADMSCs and (**C**,**D**) adipocytes derived from visADMSCs. The analysis was performed based on n = 8 different samples per group. Obese (−): adipocytes differentiated from ADMSCs of patients with obesity but without metabolic syndrome, obese (+): adipocytes differentiated from ADMSCs of patients with obesity with accompanying metabolic syndrome.

Statistical analysis was performed with R (ver. 4.3.2), which is a programming language designed for statistics and data visualization. The choice between the (non-) parametric tests was made based on the results of the Fligner–Killeen (assessment of homogeneity of variances) and Shapiro–Wilk (assessment of normality) tests. We employed a three-way ANOVA (factors: tissue, obesity and metabolic syndrome). In addition to the above, within-tissue comparisons were made with Student’s *t*-test or Wilcoxon rank-sum test. The data were displayed as bar plots (Figure 1, Figure 2, Figure 3, Figure 4 and Figure 5) with the mean and standard deviation used as the measures of concentration and dispersion, respectively. The Pearson correlation coefficients were used to examine the association between the levels of cytokines in the cell medium. The results were displayed using heatmaps (Figure 6). Logistic regression was employed as a simple method for initial feature selection (Table 1), whereas principal component analysis (Figure 7) was used as a dimensionality reduction and clustering technique. The *p*-values ≤ 0.05 were considered to be statistically significant.

## 3. Results

### 3.1. Interleukins Concentrations

We examined the expressions of the following interleukins: IL-1β, IL-1ra, IL-2, IL-4, IL-5, IL-6, IL-7, IL-8, IL-9, IL-10, IL-12, IL-13, IL-15 and IL-17. Virtually all of the examined molecules had significantly greater expressions in the adipocytes derived from ADMSCs of visceral origin when compared with their subcutaneous counterparts. The differences were in the range of roughly 2–3 folds for IL-5, IL-6 (Figure 1E,F) and IL-9 (Figure 2C) up to ~40 fold for IL-4 (Figure 1D). Of note, in the case of the cells of subcutaneous provenance, the concentrations of IL-1ra (Figure 1B) and IL-15 (Figure 2G) were below the lower level of detection (~13 pg/mL and ~10 pg/mL, respectively) and their exact levels could not be precisely determined. Therefore, although we may safely report the between-tissue differences, we were unable to establish the effect of obesity and metabolic syndrome on the secretion of interleukins in these adipocytes. Importantly, IL-8 was the only interleukin that displayed a higher concentration in the medium obtained from the cells derived from subADMSCs (+36%, *p* < 0.05, Figure 2B) when compared with the groups of visADMSC provenance.

The three-way ANOVA revealed a systematic effect exerted by obesity on the concentrations of some of the examined interleukins. Importantly, the condition was associated with lower levels of IL-1β (−0.66 pg/mL), IL-2 (−3.42 pg/mL), IL-4 (−7.72 pg/mL), IL-12 (−1.88 pg/mL), IL-13 (−1.13 pg/mL) and IL-17 (−8.71 pg/mL) (Figure 1 and Figure 2). The changes were especially pronounced in the cells of visADMSC origin. Interestingly, the level of interleukin 6 was significantly greater in the cells derived from the ADMSCs of obese patients (+319 pg/mL, Figure 1F). Of note, metabolic syndrome was also associated with lower levels of IL-2 (−2.81 pg/mL, Figure 1C), IL-7 (−17.7 pg/mL, Figure 2A) and IL-10 (−2.18 pg/mL, Figure 2D).

### 3.2. Growth Factors Concentrations

The concentration of FGF was below the lower level of detection (~3 pg/mL), but only in the cells of subADMSC provenance (Figure 3B). Of the examined molecules, the RANTES concentration was relatively stable and displayed no apparent pattern (Figure 4D). Most of the remaining proteins, i.e., VEGF, G-CSF, GM-CSF and TNF-α, were secreted to a greater extent from the cells derived from ADMSCs of visceral ancestry, with the difference in the range of +60% (VEGF, Figure 3F) to ≥ 400% (FGF, Figure 3B). Regarding VEGF, its amount was comparable in the cells that stemmed from lean patients (subLean ≈ visLean), and it grew in the adipocytes derived from the ADMSCs of obese patients. Of note, in the case of subcutaneous tissue, metabolic syndrome led to a lower expression of VEGF (−33% and −40% for subObese (+) vs. subLean and subObese (+) vs. subObese (−), respectively; *p* < 0.05; Figure 3F). However, in the case of the cells of visceral provenance, both obesity and metabolic syndrome led to a greater concentration of VEGF in the medium (+48% for visObese (−) vs. visLean, *p* < 0.05, and +123% and +51% for visObese (+) vs. visLean and visObese (+) vs. visObese (−), *p* < 0.05; Figure 3F). On the other hand, obesity was associated with a smaller concentration of FGF (−51% and −68% for visObese (−) vs. visLean and visObese (+) vs. visLean, *p* < 0.05; Figure 3B) and GM-CSF in the medium (on average by −0.43 pg/mL, *p* < 0.05; Figure 3D).

### 3.3. Chemokines Concentrations

Except for MIP-1α and MCP-1, whose levels were relatively stable (Figure 4A,C), all of the examined chemokines displayed a clear tissue-specific pattern. The adipocytes differentiated from visADMSCs secreted more chemokines to the medium when compared with their counterparts of subADMSC provenance, with the average difference in the range of ~2–3 folds. This held true for IP-10, MIP-1β and INF-γ (Figure 3E and Figure 4B,F). However, a striking difference of a few hundred folds was observed for Eotaxin (Figure 3A). Importantly, obesity led to a lower expression of Eotaxin (on average by −663 pg/mL). On the other hand, both obesity and metabolic syndrome were characterized by an increased level of IP-10 (on average by 340 pg/mL and 592 pg/mL, respectively; Figure 3E). The former was especially pronounced in the cells obtained from visADMSCs obtained from patients with obesity and accompanying metabolic syndrome (+137% and +176% for visObese (+) vs. visLean and visObese (−), respectively; *p* < 0.05; Figure 3E).

For ease of interpretation, we summarized the results presented in Figure 1, Figure 2, Figure 3 and Figure 4 into a Venn diagram (Figure 5).

### 3.4. Correlation Analysis

We conducted a separate analysis for the association between the analyzed variables with respect to the cells’ tissue of origin and metabolic status of the donor patient. The results are presented in the form of heatmaps and depicted in Figure 6. To control for the false discovery rate, we employed a *p*-value multiplicity correction (Benjamini–Hochberg procedure).

Regarding the cells of subcutaneous provenance, we observed an increased percentage of positive associations between the investigated variables in the cells derived from ADMSCs of patients with obesity and metabolic syndrome (8/0, 11/1 and 74/0 of positive/negative Pearson correlation coefficients (*p* < 0.05) for subLean, subObese (−) and subObese (+), respectively; Figure 6). Of the analyzed molecules, three associations were observed in all three groups, irrespective of the donor patient metabolic status, namely, IL-9 vs. RANTES (r = 0.92–0.96, *p* < 0.05), IL-5 vs. IL-9 (r = 0.92–0.99, *p* < 0.05), MIP-1β vs. IL-9 (r = 0.92–0.98, *p* < 0.05) and IL-6 vs. IL-9 (r = 0.92–0.96, *p* < 0.05). Moreover, visual inspection of Figure 6A–C indicates that in all of the groups, IL-10 (and sometimes IL-12) was negatively correlated with the remaining variables. However, the abovementioned pattern usually failed to reach the level of statistical significance (adjusted *p*-values > 0.05).

Similarly, the culture medium obtained from the cells derived from visADMSCs also displayed a greater percentage of positive correlations between the cytokines; however, in this case, it was visible in both the visObese (−) and visObese (+) groups (9/0, 54/1 and 41/0 of positive/negative Pearson correlation coefficients (*p* < 0.05) for visLean, visObese (−) and visObese (+), respectively; Figure 6D–F). Only, two variables (IL-5 and IL-9) were positively correlated in all the analyzed groups (r = 0.96–0.98, *p* < 0.05). On the other hand, in the two obese groups [visObese (−) and visObese (+)], we observed 21 positive correlations, namely, IL-1β vs. IL-4 (r = 0.9–0.98, *p* < 0.05), IL-1β vs. IL-13 (r = 0.88–0.98, *p* < 0.05), IL-1β vs. IL-17 (r = 0.9–0.99, *p* < 0.05), IL-1β vs. TNF-α (r = 0.95–0.96, *p* < 0.05), IL-1β vs. GM-CSF (r = 0.93–0.97, *p* < 0.05), IL-1ra vs. IL-7 (r = 0.88, *p* < 0.05), IL-4 vs. IL-17 (r = 0.91–0.98, *p* < 0.05), IL-4 vs. Eotaxin (r = 0.9–0.97, *p* < 0.05), IL-4 vs. GM-CSF (r = 0.94–0.96, *p* < 0.05), IL-5 vs. IL-9 (r = 0.94–0.96, *p* < 0.05), IL-5 vs. Eotaxin (r = 0.92–0.93, *p* < 0.05), IL-7 vs. IL-10 (r = 0.86–0.94), IL-7 vs. IL-12 (r = 0.91–0.94, *p* < 0.05), IL-9 vs. Eotaxin (r = 0.91–0.97, *p* < 0.05), IL-10 vs. IL-12 (r = 0.88–0.97, *p* < 0.05), IL-13 vs. IL-17 (r = 0.89, *p* < 0.05), IL-13 vs. TNF-α (r = 0.94–0.97, *p* < 0.05), GM-CSF vs. IL-17 (r = 0.93, *p* < 0.05), GM-CSF vs. Eotaxin (r = 0.86–0.93, *p* < 0.05), GM-CSF vs. G-CSF (r = 0.9–0.91, *p* < 0.05) and GM-CSF vs. TNF-α (r = 0.91–0.93, *p* < 0.05).

### 3.5. Logistic Regression Analysis

We expected that the development of a fully functional logistic regression model for categorization of the culture medium of cells with respect to the donor patients’ metabolic status would not be possible. The above was due to a relatively small sample size and numerous strong positive correlation coefficients observed between the examined variables (see Figure 6). Still, we decided to employ the technique as a relatively simple screening method that allowed for exploring possible prognostic variables that could serve as biomarkers for the feature of interest (here, obesity and metabolic syndrome). The minimal adequate model was constructed using forward stepwise regression, as described by Altman [20]. The results of the conducted analyses are summarized in Table 1.

With respect to the cells of subADMSC origin, our analysis demonstrated that the subObese (+) group was separated from the other two groups [subLean and subObese (−)]. It was not possible to clearly indicate the best set of predictors, e.g., due to the positive correlations between the variables (see Figure 6). However, the concentrations of the following proteins could be a reasonable initial choice, as they allowed for disassociating subObese (+) from the other two groups: IL-7, IL-10, IL-13 and VEGF (Table 1). This was in line with the previously discussed results depicted in Figure 3 and Figure 4, as the levels of all the before-mentioned variables were distinctively lower in the cells from the subObese (+) group.

The picture that stems from the analysis (Table 1) is different and somewhat less clear in the case of the secretome of adipocytes of visADMSC ancestry. In general, none of the candidate variables allowed for separation between the two obese groups [visObese (−) vs. visObese (+)], as they appeared to be similar with regard to their cytokine profiles. On the other hand, both the obese groups differed from visLean (Table 1). There were no common predictor variables for both the cases. The separation between visLean and visObese (−) could be performed based on the FGF concentration, which was significantly higher in the cells derived from the ADMSCs of lean patients (Figure 3B). On the other hand, the variables that allowed for distinguishing visLean and visObese (+) were IL-10, GM-CSF and TNF-α, all of which had higher levels in the culture media in the lean group. Furthermore, none of the predicates from Table 1 allowed for categorization of the cells based on their metabolic status in both the adipocytes of subADMSC and visADMSC provenances. Still, IL-10 occurred as the candidate predictor most often.

### 3.6. Principal Component Analysis

To further explore the cytokine profiles of the cells, we ran a principal component analysis (PCA) on the standardized ($\bar{x}$ = 0, sd = 1) variables. Its results are depicted in Figure 7. Briefly, in both cases (the culture media of cells derived from sub- and visADMSCs), roughly 75–80% of the data variability were contained in the first three principal components (PC1–PC3). A visual inspection of Figure 7A,B demonstrates that in the case of the cells of subcutaneous provenance, two separate clusters of points, i.e., orange and green, can be observed. The colors represent the cells from the subObese (−) and subObese (+) groups, respectively. On the other hand, as indicated by the spatial arrangement of the points in Figure 7C,D, the separation occurred between visLean (blue points) and visObese (−) (orange points) and (to a slightly smaller extent) between visLean (blue points) and visObese (+) (green points). Based on Figure 7C,D, the precise categorization between the two groups derived from visADMSCs of obese patients did not appear to be possible. The above supports the findings obtained from the logistic regression (Table 1) and the pattern found in the correlation heatmaps (Figure 6).

## 4. Discussion

The main goal of our investigation was to thoroughly examine the secretory profile of the adipocytes derived from ADMSCs. Particularly, we were interested to know whether the newly derived adipocytes reflected the cytokine secretory profile of the donor patients (lean, obese with/without metabolic syndrome) that could be found in the literature [8]. The existence of a fat-depot-specific pattern was previously established in a study conducted by Kahn et al. [21], namely, visceral tissue tends to release more cytokines to their surrounding environment than its subcutaneous counterpart. This characteristic was especially evident for IL-13, IL-12, IL-8, IL-6 and TNF-α [21], the last three of which displayed the highest concentrations. Moreover, the authors demonstrated that a VAT-conditioned medium rich in pro-inflammatory cytokines reduced the insulin sensitivity (lower insulin stimulated glycogen synthesis and IRS1 phosphorylation) in rat primary hepatocytes [21]. The above is in line with the general literature consensus that visceral fat is highly metabolically active, and due to its location, it releases a plethora of factors into the portal circulation, which affects the liver, and thus, triggers systemic insulin resistance [8]. Interestingly, the results of Kahn and co-authors seem to also be reflected in our current study, as virtually all the investigated cytokines were present in a greater concentration in the medium that bathed the adipocytes of visADMSC provenance, where IL-6, IL-8 and TNF-α reached the highest levels (in pg/mL, Figure 1F, Figure 2B, Figure 4E and Figure 5).

However, there were also some discrepancies between our results and the ones reported by other teams with regard to the adipose tissue cytokine secretory profile. The most evident were those where general obesity was characterized, among others, by a chronic low-level inflammation. The body mass index has been reported to be positively correlated with both the serum concentration and adipose tissue expression of many cytokines. Such a phenomenon was observed, e.g., for IL-2 [22], IL-6 [23,24], IL-8 [25], TNF-α [24,26] and MCP-1 [25]. Interestingly, the results yielded by our current study are less straightforward. On the one hand, the cells derived from the ADMSCs of patients with obesity and metabolic syndrome did display a greater number of positive correlations between the cytokines than their lean counterparts (Figure 6). This points to a propensity of the cells toward an inflammatory state. On the other, the majority of the examined conventionally considered pro-inflammatory cytokines displayed a lower concentration in the culture medium when it bathed the cells derived from the ADMSCs of patients with obesity and the accompanying metabolic syndrome. It is hard to satisfactorily explain this finding since to the best of our knowledge, this is the first study that examined the secretory profile of mature adipocytes obtained from mesenchymal stem cells (ADMSCs). A sound putative justification of the observation would be the fact that while adipocytes do secrete the aforementioned cytokines, the majority of them are in fact released by the activated immune cells (e.g., macrophages) that infiltrate the tissue [8]. Weisberg and co-authors [27], for instance, assessed the number of macrophages (immunohistochemistry, F4/80-expressing cells) in the adipose tissue of female mice. The tissue obtained from lean animals presented a significantly lower percentage of the immune cells (~12% of the total cell count) when compared with the one obtained from high-fat-diet-fed animals (~41% of the total cell count) [27]. Moreover, the researchers estimated the fraction of macrophages to be below 10% and over 50% for the adipose tissue of lean and morbidly obese humans, respectively [27]. Briefly, the available literature suggests that overfeeding and a lack of physical activity leads to the accumulation of the energy surplus in the form of triacylglycerols stored in body fat [8]. At one point, the hypertrophied tissue releases a set of cytokines (among the most commonly mentioned are TNF-α, IL-1β, IL-6 and MCP-1) that act as chemoattractants for lymphocytes and monocytes [28]. The latter infiltrate the tissue and differentiate into macrophages that, in turn, secrete even more cytokines that allure even more leukocytes that produce still greater quantities of cytokines (a vicious circle powered by a positive feedback loop) [8]. In line with this notion and based on our current research, we could postulate that IL-6, IP-10 and VEGF are among the primary factors released by the adipocytes that set the whole cycle into motion. All three molecules displayed a clear few-fold increase in concentration when measured in the medium used to cultivate the cells of the visObese (+) group (Figure 1F and Figure 3E,F). Indeed, IL-6 and IP-10 are classified as pro-inflammatory cytokines characterized by the chemoattraction of monocytes/macrophages, T cells, NK cells and neutrophils, as well as the regulation of B-cell maturation [29]. However, caution needs to be applied when interpreting any cytokine data since the research often yields contradictory results. For instance, Bastard et al. [30] investigated the impact of IL-6 on the development of metabolic consequences of obesity in human subjects. The authors found a strong negative correlation (r ≈ −0.75, *p* < 0.05) between the adipose tissue IL-6 content [pg/g of tissue] and glucose uptake (both basal and insulin stimulated). The above points to the cytokine as a potential trigger of glucose metabolism disarrangements that are a hallmark of metabolic syndrome. On the other hand, a study by Matthews and co-workers [31] demonstrated that IL-6 knock-out mice were characterized by obesity, insulin resistance and hepatosteatitis, even when fed with a standard chow diet, thus yielding contradictory results. It turns out that the interleukin actions may vary depending on its target tissue. Bobbo et al. [32], for instance, demonstrated that IL-6 may protect against diet-induced body mass gain via its action on the hypothalamus (control of the neurogenesis of POMC- and NPY/AgRP-producing neurons that generate the sensation of satiety and hunger, respectively). Interestingly, studies demonstrated that the site of cytokine secretion may also determine its biological effects [33]. The two main sites of IL-6 secretion (except for the monocytes/macrophages) are adipose tissue and skeletal muscles. Han and colleagues [33] demonstrated that the response of adipose tissue differs depending on the source of the interleukin. When secreted by the adipocytes themselves, IL-6 increases macrophages infiltration of the tissue (via so called non-canonical trans-signaling mode). On the other hand, IL-6 of skeletal muscle origin (secreted, e.g., in response to physical activity) suppressed the process (via canonical myeloid cells). The above adds importance to the increase in the IL-6 concentration in the medium observed by us and its proposed role as a triggering factor for tissue inflammation.

Paradoxically, low cytokine levels may be a good reflection of the early disarrangements present in the adipocytes of obese patients. In general, chronic overfeeding leads to the deposition of superfluous lipids in the adipose tissue that undergoes hypertrophy to accommodate such an excess in energy substrates [8]. Once the storage capacity of the tissue is exceeded, the excessive FFAs overflow to the adjacent organs, like the liver, or skeletal muscle [8,14]. The stockpiling of lipids in the cells not adapted for this purpose is known as ectopic fat accumulation and is believed to directly contribute to the metabolic dysfunctions (like insulin resistance, metabolic syndrome or type 2 diabetes) found in obesity [34,35]. In line with this notion, Nov and colleagues [17] demonstrated that IL-1β-KO mice fed with a high-fat diet had more spacious fat depots than their wild-type counterparts fed with the same chow. The above was accompanied by a greater expression of adipogenesis markers (e.g., PPAR-γ, FABP4) and little to no increase in the expression of pro-inflammatory genes [17]. Interestingly, these alterations of adipose tissue phenotype were correlated with a lower liver mass, smaller steatosis and maintained insulin sensitivity of the organ [17]. Thus, the authors suggested that IL-1β promotes ectopic fat deposition in hepatocytes, which, in turn, leads to the exacerbation of the metabolic syndrome observed in obesity [17]. Importantly, other teams also postulated the existence of a metabolic switch between the lipid storage and inflammatory phenotype in adipocytes [36,37]. The theory is a reasonable explanation of the results that stem from this study (a drop in pro-inflammatory cytokine secretion, e.g., IL-1β in Figure 1A, found in the medium surrounding the cells from ADMSCs of obese patients with metabolic syndrome). Moreover, the notion is also supported by our recent research [14] conducted on the same cell model. Previously, we demonstrated that the adipocytes derived from the ADMSCs of patients denoted as obese (+) had a greater expression of fatty acid transporters (CD36/SR-B2, FATP4), more pronounced lipids (H3-palmitate) uptake and the activation of lipid synthesis pathways (↑FASN, ↑DGAT1) [14] when compared with their lean counterparts. Collectively, the data indicate that the adipocytes derived from the ADMSCs of obese patients with metabolic syndrome are activated toward the lipid accumulation profile at the expense of cytokine secretion, which is an early marker of the metabolic disarrangements that take place in adipose tissue in the course of obesity.

The current research was not without shortcomings that should be addressed by future investigations. Ours was a preliminary study on a previously unexplored topic, whose outcomes were hard to predict at the onset. Therefore, for our exploratory analyses, we chose a possibly large and representative set of cytokines. For this purpose, we deployed the broadest cytokine screening assay (27-plex) available to us at the time. Still, the estimated number of molecules that belong to the cytokine category is well over one hundred. For this reason, future studies on the topic are warranted to broaden our understanding of the cells’ secretory profiles. Moreover, other approaches, such as the investigation of the chemoattractant properties of the secreted cytokines would be a valuable addition to subsequent research. Lastly, the number of biological replicates (n = 8 per group), although common in the papers from the field, is relatively small in absolute terms. A study of greater size would allow for drawing stronger conclusions by counterbalancing between-patient data variability.

## 5. Conclusions

In summary, to date, this is the first research that thoroughly examined the cytokine secretome in the mature adipocytes derived from stem cells of lean and obese women. Our study demonstrated that the adipocytes display a characteristic secretory fingerprint when derived from the ADMSCs of patients with obesity and metabolic syndrome. Moreover, we identified a set of cytokines (IL-1β, IL-6, IL-7, IL-10, IL-12, IL-13, VEGF, FGF, GM-CSF, TNF-α, IFN-γ) that could serve as putative markers of the condition. Surprisingly, and in contrast to our initial hypothesis, the concentrations of most of the abovementioned molecules were decreased in the medium that bathed the cells descended from the ADMSCs of obese patients with metabolic syndrome. This was in contrast with the conditions observed in clinical studies since most research reported an increased amount of pro-inflammatory cytokines. Most likely, the difference stemmed from the fact that in vivo, the molecules are mostly secreted by the activated monocytes/macrophages and not the adipocytes per se. Moreover, an increased secretion of pro-inflammatory cytokines from fat tissue usually occurs when its storage capacity for lipids is surpassed. This results in the spillover of lipids into other, unadjusted for their storage, organs. This process is known in the literature as ectopic fat accumulation and is believed to be the direct cause of metabolic dysfunctions (i.e., insulin resistance, type 2 diabetes) found in obesity. Therefore, low levels of pro-inflammatory cytokines observed in this study predestine the adipocytes toward lipid accumulation and may be a hallmark of early metabolic disarrangements that occur in the tissue in the progress of obesity. Overall, this indicates that the adipocytes newly derived from the ADMSCs of obese patients with metabolic syndrome are still able to ‘buffer’ the body’s lipids surplus.

## Figures and Tables

**Table 1:** Logistic regression analyses of cytokine concentrations in the media that bathed mature adipocytes differentiated from ADMSCs.

Groups in Model	Coding of The Dependent Variable	Possible Predicate	Coefficient for Predicate	*p*-Value for a Model with a Single Predicate
subLean, subObese (−)	subLean = 1 subObese (−) = 0	None	--	--
	minimal adequate model	Y = −0.000; P(1) = $\frac{1}{1+e^{-y}}$		
subLean, subObese (+)	subLean = 1 subObese (+) = 0	IL-7	0.095	0.036
		IL-10	1.737	0.026
		IL-13	8.789	0.022
		Eotaxin	0.206	0.04
		VEGF	0.014	0.035
	Minimal adequate model	Y = −11.27 − 8.789 × IL-13 P(1) = $\frac{1}{1+e^{-y}}$		
subObese (−), subObese (+)	SubObese (+) = 1 subObese (−) = 0	IL-1B	−2.543	0.045
		IL-2	−0.766	0.041
		IL-7	−0.16	0.049
		IL-10	−1.38	0.037
		IL-12	−0.75	0.049
		IL-13	−7.25	0.039
		VEGF	−0.0098	0.044
	Minimal adequate model	y = 2.94 − 1.381 × IL-10 P(1) = $\frac{1}{1+e^{-y}}$		
visLean, visObese (−)	visLean = 1 visObese (−) = 0	FGF	0.261	0.04
	Minimal adequate model	Y = −4.69 + 0.261 × FGF P(1) = $\frac{1}{1+e^{-y}}$		
visLean, visObese (+)	visLean = 1 visObese (+) = 0	IL-10	0.525	0.0462
		GM-CSF	4.295	0.0457
		TNF-α	0.058	0.05
	Minimal adequate model	y = −9.275 + 4.295 × GM-CSF P(1) = $\frac{1}{1+e^{-y}}$		
visObese (−), visObese (+)	VisObese (+) = 1 visObese (−) = 0	None	–	--
	Minimal adequate model	y = −0.000; P(1) = $\frac{1}{1+e^{-y}}$		

## Data Availability

The data presented in this study are available on request.

## Supplementary Materials

The following supporting information can be downloaded at: https://www.mdpi.com/article/10.3390/cells13191603/s1, Figure S1. Basic characteristics of the mature adipocytes differentiated from ADMSCs [19]. Table S1. Clinical characteristics of patients (tissue donors).
