# Supplementary material for: Altered Cytokine Secretory Fingerprint of the Adipocytes Derived from Stem Cells of Morbidly Obese Patients—A Preliminary Study
Source: Cells. 2024 Sep 24;13(19):1603. doi: 10.3390/cells13191603 (PMC11475718; doi:10.3390/cells13191603)
Supplement: Supplementary file 1 [file cells-13-01603-s001.zip › cells-3207161-supplementary.pdf]

cells MDPI

Supplementary Materials

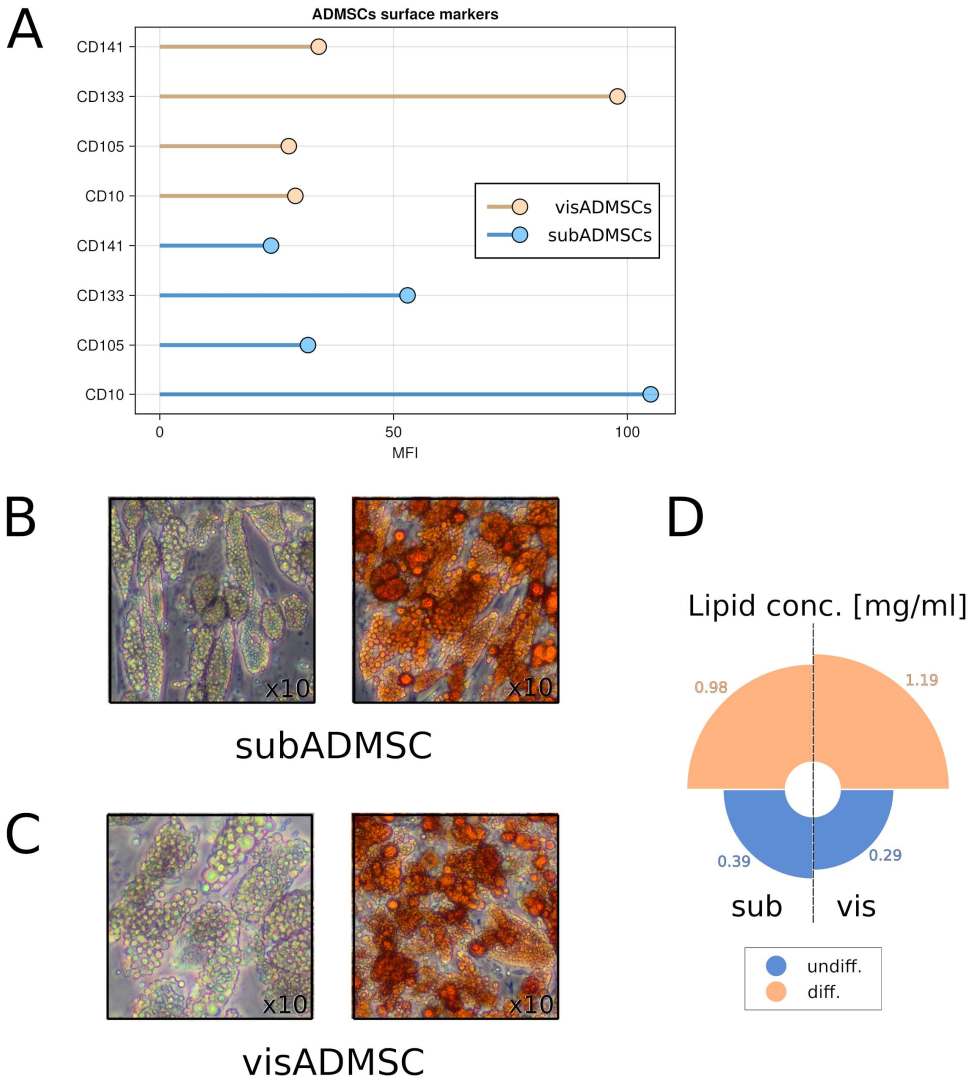

Figure S1. Basic characteristics of the ADMSCs and mature adipocytes. (A) Mean fluorescent intensity (MFI) for CD10+, CD105+, CD133+ and CD141+ markers found on adipose derived mesenchymal stem cells (ADMSCs) obtained from subcutaneous and visceral fat depots (as reported in [19]). (B) Mature adipocytes differentiated from ADMSCs of subcutaneous fat depots under light microscopy. Clearly visible lipid droplets (right panel dyed with Oil Red O Staining). (C) Mature adipocytes differentiated from ADMSCs of visceral fat depots under light microscopy. Clearly visible lipid droplets (right panel dyed with Oil Red O Staining). (D) Quantification of lipid content in the un- and differentiated ADMSCs (as reported in [19]).

Table S1. Clinical characteristics of patients (tissue donors).

	**Lean**	**Obese(-)**	**Obese(+)**
age [years]	59.5 ± 3.5	59.8 ± 2.9	60.5 ± 3.3
glucose [mg/dL]	76.2 ± 7.8	98.2 ± 12.0 a	104.8 ± 8.3 a
CRP [mg/L]	5.6 ± 0.4	9.3 ± 0.8 a	12.4 ± 2.3 ab
WHR	0.8 ± 0.13	0.9 ± 0.03	0.9 ± 0.02 a
BMI [kg/m^2]	24.1 ± 2.6	42.4 ± 2.1 a	50.1 ± 4.2 ab
Insulin [uIU/mL]	7.3 ± 0.7	20.2 ± 2.5 a	27.8 ± 6.6 ab
LDL [mg/dL]	111.2 ± 4.0	120.0 ± 9.3 a	136.8 ± 33.5 ab
TAG [mg/dL]	140.5 ± 4.8	147.5 ± 6.3 a	173.0 ± 17.9 ab
HDL [mg/dL]	59.8 ± 5.8	49.8 ± 4.0 a	37.5 ± 11.5 ab
SP [mmHg]	120.0 ± 9.6	118.8 ± 10.3	148.8 ± 10.3 ab
DP [mmHg]	78.8 ± 4.4	85.2 ± 4.7 a	93.8 ± 4.4 ab
WBC [10^3/μL]	7.9 ± 1.4	7.9 ± 1.5	7.8 ± 1.6
RBC [10^3/μL]	4.8 ± 0.1	4.6 ± 0.4	4.9 ± 0.3
HGB [g/dL]	14.3 ± 1.1	13.9 ± 0.9	13.3 ± 0.8
PLT [10^3/μL]	291.1 ± 17.46	282.2 ± 47.01	291.1 ± 25.4

Data are presented as mean +/- SD. $P < 0.05$; a – indicate significant differences from lean subjects; b – indicate significant differences from Obese(-) group. BMI – body mass index; CRP – C-reactive protein; DP – diastolic pressure; HDL – high density lipoprotein; HGB – hemoglobin; LDL – low-density lipoprotein; PLT – platelet count; RBC – red blood cell count; SP – systolic pressure; TAG – triacylgycerol; WBC – white blood cell count; WHR – waist-hip ratio.
